# Self-Actuated Paper and Wood Models: Low-Cost Handcrafted Biomimetic Compliant Systems for Research and Teaching

**DOI:** 10.3390/biomimetics6030042

**Published:** 2021-06-22

**Authors:** Simon Poppinga, Pablo Schenck, Olga Speck, Thomas Speck, Bernd Bruchmann, Tom Masselter

**Affiliations:** 1 Plant Biomechanics Group @ Botanic Garden, University of Freiburg, 79104 Freiburg im Breisgau, Germany; pabloschenck7@yahoo.de (P.S.); olga.speck@biologie.uni-freiburg.de (O.S.); thomas.speck@biologie.uni-freiburg.de (T.S.); 2 Freiburg Materials Research Center (FMF), University of Freiburg, 79104 Freiburg im Breisgau, Germany; 3 Cluster of Excellence livMatS @ FIT—Freiburg Center for Interactive Materials and Bioinspired Technologies, University of Freiburg, 79110 Freiburg im Breisgau, Germany; 4 BASF SE, Advanced Materials and Systems Research, 67056 Ludwigshafen/Rhein, Germany; bernd.bruchmann@basf.com

**Keywords:** actuators, biomimetics, compliant systems, hygroscopic materials, plant movements

## Abstract

The abstraction and implementation of plant movement principles into biomimetic compliant systems are of increasing interest for technical applications, e.g., in architecture, medicine, and soft robotics. Within the respective research and development approaches, advanced methods such as 4D printing or 3D-braiding pultrusion are typically used to generate proof-of-concept demonstrators at the laboratory or demonstrator scale. However, such techniques are generally time-consuming, complicated, and cost-intensive, which often impede the rapid realization of a sufficient number of demonstrators for testing or teaching. Therefore, we have produced comparable simple handcrafted compliant systems based on paper, wood, plastic foil, and/or glue as construction materials. A variety of complex plant movement principles have been transferred into these low-cost physical demonstrators, which are self-actuated by shrinking processes induced by the anisotropic hygroscopic properties of wood or paper. The developed systems have a high potential for fast, precise, and low-cost abstraction and transfer processes in biomimetic approaches and for the “hands-on understanding” of plant movements in applied university and school courses.

## 1. Introduction

### 1.1. Motivation

Although they do not possess muscles, nerves, and “typical” (technical rotating) hinges, plants can perform a variety of complex motions. The speed of movement spans several orders of magnitude and depends on the dimension of the motile plant structure, its mechanical properties, and the actuation principle [1]. Whereas slow motion is typically driven by hydraulic processes, i.e., water displacement processes between cells and tissues, fast motion is characterized by (additional) elastic instabilities, i.e., the release of elastic energy accumulated and stored in a deformed (prestressed) structure. Hydraulics include turgor changes, which require metabolic energy during the motion, and energetically cost-free hygroscopic swelling/shrinking processes (reviewed by [2,3,4]). Famous examples of plant movements are the touch-sensitive leaflets and leaves of *Mimosa pudica*, which move by turgor changes [5], the hygroscopic opening and closing of pine cones (*Pinus* spp.) [6], and the snapping of the carnivorous Venus flytrap (*Dionaea muscipula*), which incorporates both turgor changes and the release of prestress [7,8].

Motile plant structures change shape during movement and, therefore, constitute compliant systems [9,10]. This is in contrast to rigid-body mechanisms with “classical” hinges, as characterized by stiff structural components that glide against each other and that are typically connected by rotating hinges. Motile plant structures are of increasing interest for biomimetic approaches in which hinge-free and functionally resilient and robust compliant mechanisms are desired, e.g., for applications in architecture, soft robotics, and medicine [11,12,13,14]. Previously existing biomimetic motile technical structures incorporate nastic responses, which involve movement in a morphologically predetermined (structurally preprogrammed) manner after being triggered [15], or tropistic reactions in which the movement direction depends on the direction of the triggering stimulus [16]. The seed scale of the pinecone is a widely employed biological concept generator for nastic biomimetic motion. Its functional bilayer architecture, which consists of tissue layers with different passive swelling and shrinking properties [6,17,18], has been abstracted and transferred into numerous motile technical materials systems. These biomimetic structures can react with bending to various stimuli, e.g., changes in humidity, temperature, pH, or light, depending on the materials used for bilayer construction [19,20,21]. By tailoring the materials and their compositions and the architecture of the material layers, more complex motions can be achieved, such as flat-to-helical transitions and coiling [22,23]. Three-dimensional printing of shape-changing structures (termed 4D printing) has become an emerging field [24,25,26], allowing the transfer of complex plant deformation principles such as multi-phase motion and curved-fold bending into 4D prints [27,28].

However, 3/4D printing and other manufacturing methods are costly, complicated, and time-consuming, all of which might hinder the rapid realization of a sufficient number of demonstrators for testing or for teaching students. Therefore, we have conducted a search for a method for the construction of low-cost, biomimetic, self-actuated compliant systems out of common, cost-effective materials. These systems should be capable of performing physically correct nastic plant deformation and motion sequences. In prior studies, we have presented handcrafted structures that mimic the movement of the bird of paradise flower (*Strelitzia reginae*) [29] and the trap movements of various carnivorous plants [30]. However, these systems are actuated extrinsically by the application of mechanical force (e.g., by manual bending) and are, therefore, not self-actuated. For the new self-actuated structures presented in this study, we have aimed at implementing passive hydraulic actuation as the main driver. Therefore, we have chosen paper and wood as basic construction materials, as these are characterized by pronounced swelling/shrinking capabilities upon hydration/dehydration [31,32]. The use of connected wood and/or paper strips with different fiber orientations to generate hygroscopically driven bending and curling motions has a long history and has also found applications in didactic approaches [33]. However, spatially complex movements have not been achieved to date. Our goal has been to transfer the deformation principles of various plant concept generators into simple and low-cost demonstrators.

### 1.2. Plant Concept Generators

#### 1.2.1. Flower of the Bird of Paradise (*Strelitzia reginae*)

**Figure 1:**
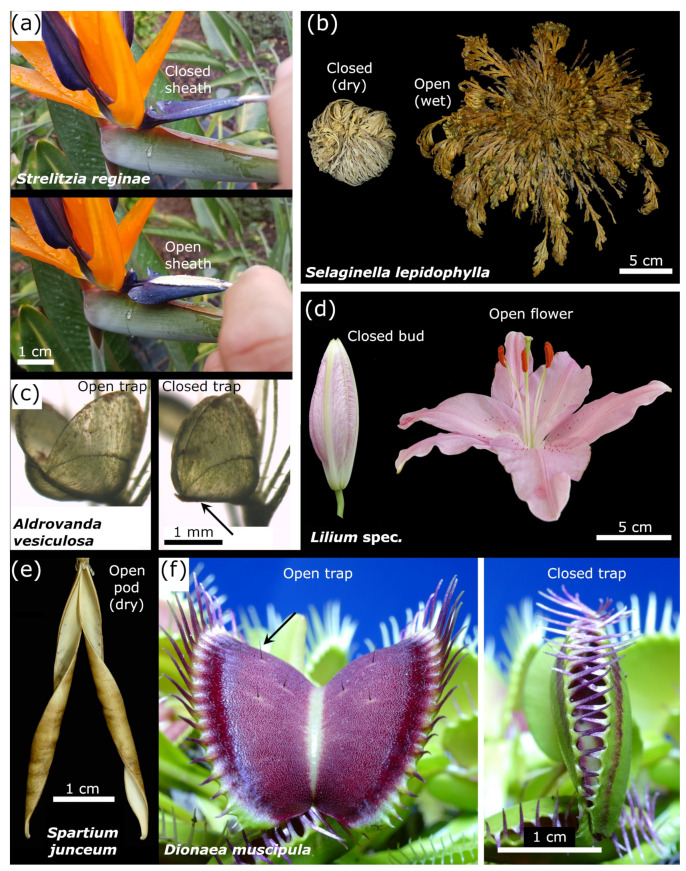
Biological concept generators for the compliant systems presented in this study. (**a**) The flower of the bird of paradise (*S. reginae*) features a motile sheath that opens upon application of a mechanical load. (**b**) The false rose of Jericho (*S. lepidophylla*) is in a strongly folded configuration when dry and unfolds during wetting. (**c**) The snap-trap of the carnivorous aquatic waterwheel plant (*A. vesiculosa*) closes after mechanical triggering of sensitive hairs inside the trap (not visible). The two trap lobes are connected by a midrib (arrow) and close because of the slight bending of the midrib but without curvature changes of the two trap lobes. Image modified after [37]. (**d**) The lily (*L.* spec.) flower opens via edge-growth-based actuation of the individual petals. (**e**) The open seed pod of the Spanish broom (*S. junceum*) is an example of the hygroscopic seed pod of members of the Fabaceae. (**f**) The snap-trap of the carnivorous Venus flytrap (*D. muscipula*) closes after the mechanical triggering of sensitive hairs (black arrow), leading to a swift concave-convex curvature change of the two trap lobes.

The flower of the bird of paradise (*S. reginae*) (Figure 1a) is characterized by a motile perch-like sheath that plays a crucial role in pollination [34,35]. When a bird lands on the sheath, which consists of two connected petals forming a cylinder open on one longitudinal side, to search for nectar at the flower base, its body weight causes a downward bending and the simultaneous sideways flapping of the two petals by torsional buckling [36]. Thereby, the previously hidden stamens are exposed, and the pollen can stick to the bird’s feet for transport to and pollination of another flower. When the bird flies away, the sheath closes, and the stamens with pollen are protected by the sheath once again. The deformation principle (torsional buckling) has been abstracted and implemented in the biomimetic façade shading Flectofin^®^ [36]. The sheath movement is reversible and highly repeatable, and is not self-actuated. In our approach, which we present here, we have aimed at producing a self-actuated technical “counterpart” of the deformation behavior.

#### 1.2.2. False Rose of Jericho (*Selaginella lepidophylla*)

The false rose of Jericho (*S. lepidophylla*) is a spike-moss capable of withstanding severe droughts and pronounced water loss (Figure 1b). Under dry environmental conditions, each of its stems is curved inward, and the whole plant has a spherical appearance [38]. Under wet conditions, the stems and leaves soak up water and unfold. The reversible hygroscopic folding/unfolding takes place in a coordinated manner, with the individual stems reacting as individual elements and is dictated by the bilayer architecture of the stems [39]. Here, our aim has been to develop a paper-based composite structure consisting of individual hygroscopic elements (the “stems”) that act as a functional unit and induce the global opening and closing response of the whole artificial “plant”.

#### 1.2.3. Snap-Trap of the Carnivorous Waterwheel Plant (*Aldrovanda vesiculosa*)

The aquatic carnivorous waterwheel plant (*A. vesiculosa*) develops snap-traps that are approximately 5 mm in length, and that can capture small aquatic prey such as water fleas (Figure 1c) [40]. The trap consists of two convex (seen from the outside) lobes that are connected by a midrib. Once prey triggers small sensitive hairs on the inner surfaces of the lobes, the trap closes within ca. 50 ms. Trap actuation involves turgor changes in motor cells and the release of prestress by downward bending (i.e., relaxation) of the prestressed midrib [40,41]. When the midrib flexes, the attached lobes are synchronously drawn together, and the trap shuts by a process called motion amplification [42]. This deformation behavior has been abstracted and implemented in the biomimetic façade shading Flectofold [43]. In the present work, we have attempted to develop artificial “traps” showing the complex coupled bending response involving motion amplification.

#### 1.2.4. Blooming of the Lily Flower (*Lilium* spec.)

The opening of the lily flower (*L.* spec.) (Figure 1d) incorporates the bursting of the bud and, subsequently, the outward curving of the petals. Instead of differential growth processes at the adaxial (=upper) and abaxial (=lower) surfaces, the actuation principle of the individual petal is based on growth-induced cell elongation processes on the petal edges (and not on the central part), finally leading to wrinkled petal edges [44]. Our objective has been to transfer this edge-actuation into abstracted artificial paper-based “petals” that are capable of curvature changes similar to those of the natural petals.

#### 1.2.5. Opening of the Legume Seed Pod

The chiral opening of the legume fruits (legume or seed pod) of many members of the Fabaceae (Figure 1e, exemplarily shown by *Spartium junceum*) is driven by shrinking processes of hygroscopic tissues. The initially straight valves of the fruit dry out, the occurring stresses lead to the rapid opening (popping) of the whole seed pod (thereby entailing ballistic seed release) [45], and the valves then slowly curl into helical strips of opposite handedness [46]. The structural bases for this flat-to-helical transition are the two fibrous layers that are found within each pod valve and that are oriented at ca. 45° with respect to the longitudinal axis of the pod. Upon drying out, the layers induce the observed deformation. The aim of this part of the project has been to transfer the basic functional morphology of the seed pod into a paper model incorporating the initial popping of the pod and the subsequent valve curling.

#### 1.2.6. Snap-Trap of the Carnivorous Venus Flytrap (*Dionaea muscipula*)

The carnivorous Venus flytrap (*D. muscipula*) develops aerial traps of ca. 2 cm in length for the capture of arthropod prey. The triggering of sensitive hairs on the internal surface of the trap by prey causes the fast shutting of the trap within 100–500 ms. The snapping process is driven by turgor changes, the release of prestress, and a sudden concave-convex geometrical change of the individual lobes [3,4]. The goal here has been to develop a compliant system capable of hygroscopically induced snap-through behavior, thereby transferring the “speed-boosting” snap-through transition of the natural snap-trap.

## 2. Materials and Methods

We chose desiccation-driven motion actuation in all of our models. This type of actuation is, from a practical viewpoint, more readily achievable (compared with a water uptake and swelling-based mechanism) and offers a broader design space since the resulting constructions do not have to be placed into humid environments (e.g., water basins or chambers with high relative humidity) for the induction of swelling and movement. The motions of all biological concept generators were abstracted and transferred into paper-based models, except for the Venus flytrap (*D. muscipula*) model, for which a paper-polymer sheet combination was used. For the snap-trap of *A. vesiculosa*, a wood-polymer-based constructional approach was chosen additionally to the paper-based model. The models were photographed, and their motions were recorded with a Panasonic Lumix DMC-FZ 1000 bridge camera.

### 2.1. Paper-Based Models

We used “PERGA pastel” paper (weight: 100 g/m^2^; Artoz Papier AG, Lenzburg, Germany) for the actuating layers (AL) and the construction paper “Tonpapier Weiß” (weight: 130 g/m^2^; L. Jansen GmbH & Co. KG, Mönchengladbach, Germany) for the resistance layers (RL). We cut both types of paper (AL and RL) with scissors according to the schemes presented in the following.

The fiber orientations of each type of paper dictate the movement responses of the envisaged models and can be perceived macroscopically or with a magnifying glass. When the paper is torn, a smooth tear is produced in the fiber orientation of the paper, whereas the tear against the fiber direction is frayed and ragged.

For construction, we initially put the ALs in a water tank for approximately 5 min until they were fully soaked. Simultaneously, we coated the RLs with a thin film of UHU Max Repair glue (UHU, Bühl, Germany) at the respective connection areas, as indicated in the schemes presented in the following. We then carefully connected the two layers and placed them in a dry environment. The ALs shrank upon desiccation, which, in combination with the resistive properties of the RLs, dictated the deformation and movements of the models.

#### 2.1.1. Flower of the Bird of Paradise (*S. reginae*)

**Figure 2:**
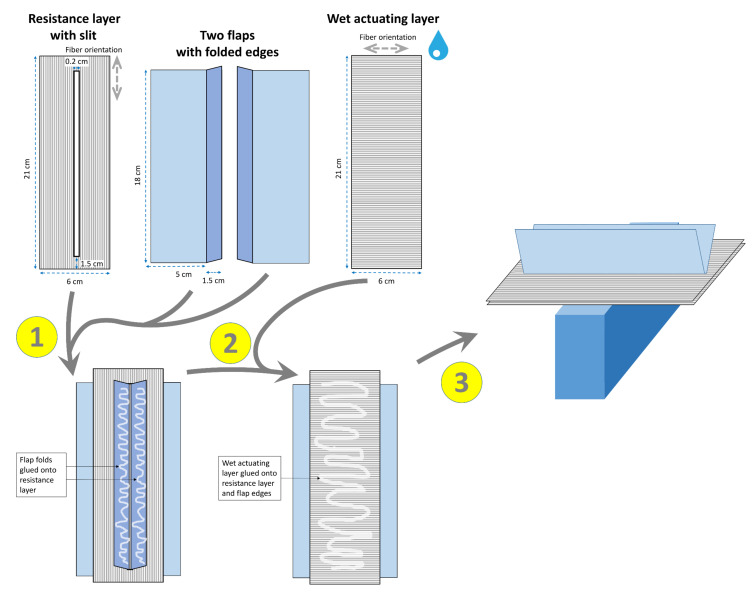
Exemplary construction scheme for the abstraction of the torsional buckling-induced bending deformation of the flower sheath of the bird of paradise (*S. reginae*) and its transfer into a paper-based self-actuated compliant system. The various construction steps are numbered (1, 2, 3). The fiber orientations of the dry RL and wet AL are indicated (gray dashed arrows). Details are provided in the main text.

The torsional buckling principle, as present in the flower sheath of *S. reginae*, can be transferred into a paper model via the construction steps depicted in Figure 2. A rectangular piece of paper forming the RL with a central slit and lengthwise fiber orientation, two paper flaps of any fiber orientation with folded edges (i.e., copy paper), and a rectangular wet AL with a transverse fiber arrangement are required. The folded edges of the flaps are inserted through the slit in the RL and glued onto its lower surface. The wet AL is then also glued onto this lower surface. The whole model is then set up in a dry environment, with the flaps oriented upwards, onto a narrow structure allowing the bending of the AL/RL bilayer.

#### 2.1.2. False Rose of Jericho (*S. lepidophylla*)

**Figure 3:**
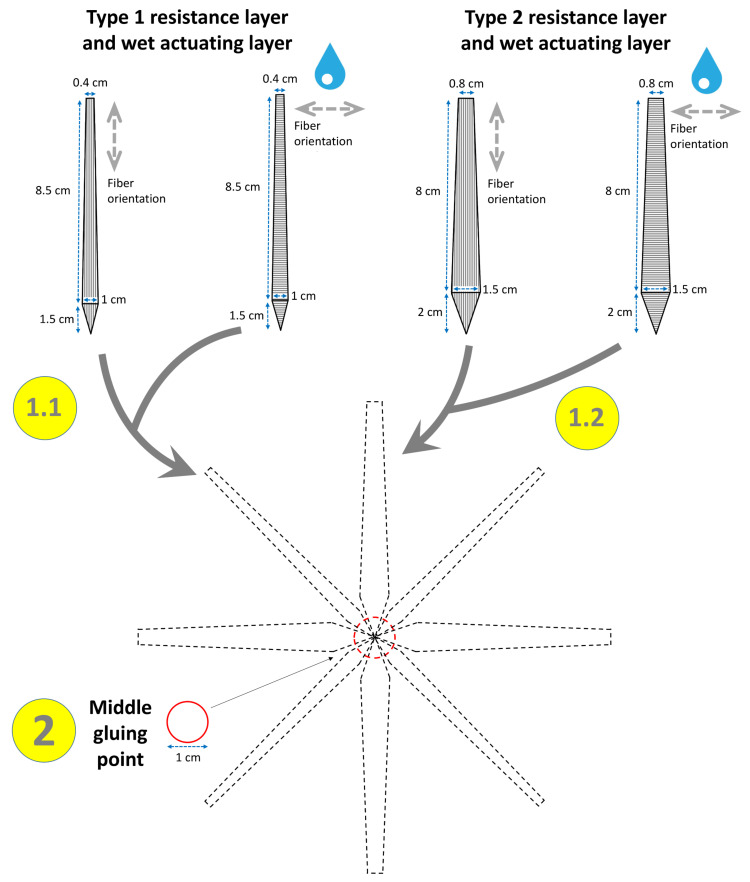
Exemplary construction scheme for the abstraction of the composite structure of the spike-moss *S. lepidophylla* and its transfer into a paper-based self-actuated compliant system. The various construction steps are numbered (1, 2). Steps 1.1 and 1.2 (construction of “stems” and their attachment by glue to the circular middle point) do not have to be undertaken in any particular order and can be repeated as many times as required (in our case: four each). The fiber orientations of the dry RLs and wet ALs are indicated (gray dashed arrows). Details are provided in the main text.

The composite architecture as present in the natural *S. lepidophylla* can be abstracted and transferred into a paper model via the construction steps depicted in Figure 3. First, a small circular middle gluing point is cut out of paper, regardless of which type of fiber orientation. Subsequently, numerous individual bilayer “stems” are produced that consist of dry RLs with lengthwise fiber orientations glued to identically shaped wet ALs with transverse fiber orientations. The “stems” are finally glued to the middle point.

#### 2.1.3. Snap-Trap of the Carnivorous Waterwheel Plant (*A. vesiculosa*)

**Figure 4:**
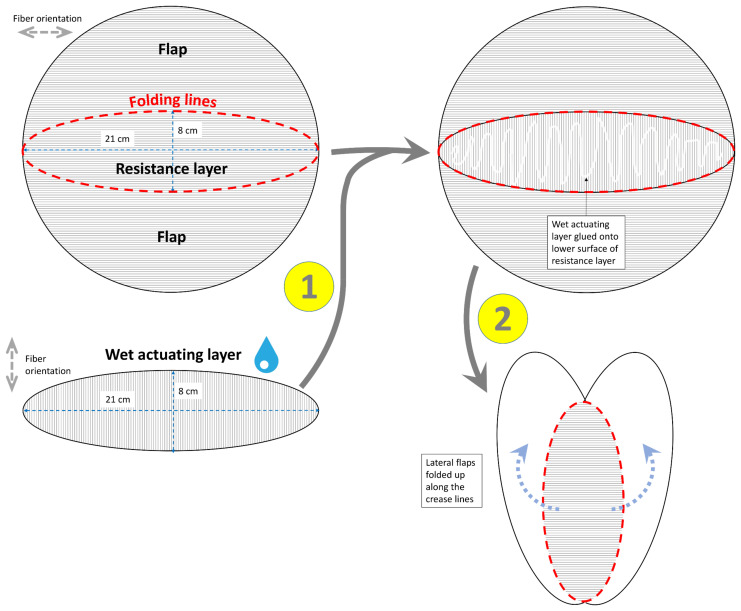
Exemplary construction scheme for the abstraction of the *A. vesiculosa* snap-trap and its transfer into a paper-based self-actuated compliant system. The various construction steps are numbered (1, 2). The fiber orientations of the dry RL and wet AL are indicated (gray dashed arrows). Details are provided in the main text.

Curved-fold bending, as present in the natural trap of *A. vesiculosa*, can be abstracted and transferred into a paper model via the construction steps depicted in Figure 4. First, a circular piece of paper is folded in such a way that an elliptical middle lens is created, representing the midrib of the natural trap and constituting the RL in our model. The fiber orientation of the paper should lie along the length of this lens. The wet AL, which is of the identical elliptical shape as the RL (lens) but with transverse fiber orientation, is glued onto the underside. The remaining parts of the round paper are folded upwards along the crease lines and, thereby, become the convex (as seen from the outside) lateral flaps. During this folding procedure, the lens becomes slightly arched.

#### 2.1.4. Blooming of the Lily Flower (*L.* Spec.)

**Figure 5:**
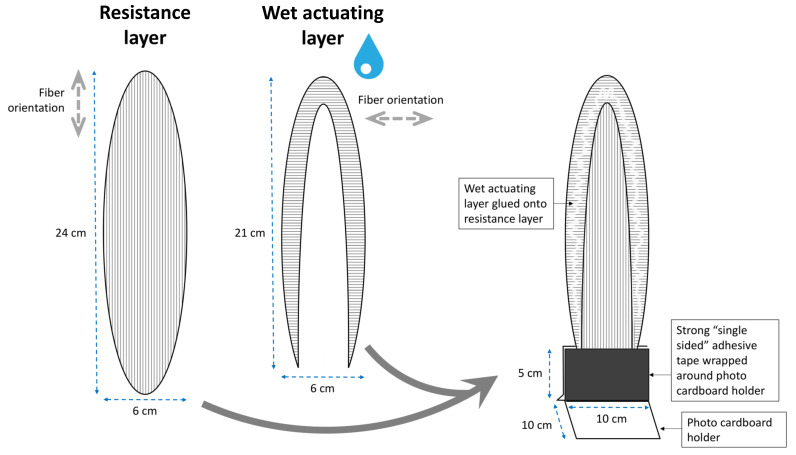
Exemplary construction manual for the abstraction of the deformation of the petal of the lily (*L.* spec.) and its transfer into a paper-based self-actuated compliant system. The fiber orientations of the dry RL and wet AL are indicated (gray dashed arrows). Details are provided in the main text.

The petal deformation principle of the lily (*L.* spec.) can be abstracted and transferred into a paper model via the construction steps depicted in Figure 5. First, an elliptical RL is cut out having a lengthwise fiber orientation. For the AL, another ellipse of identical shape but with a transverse fiber orientation is created and then cut in such a way that only the upper margin remains. This margin is then glued onto the RL, thereby creating a bilayer zone. For convenience, we also constructed a holder made from cardboard and adhesive tape.

#### 2.1.5. Opening of the Legume

**Figure 6:**
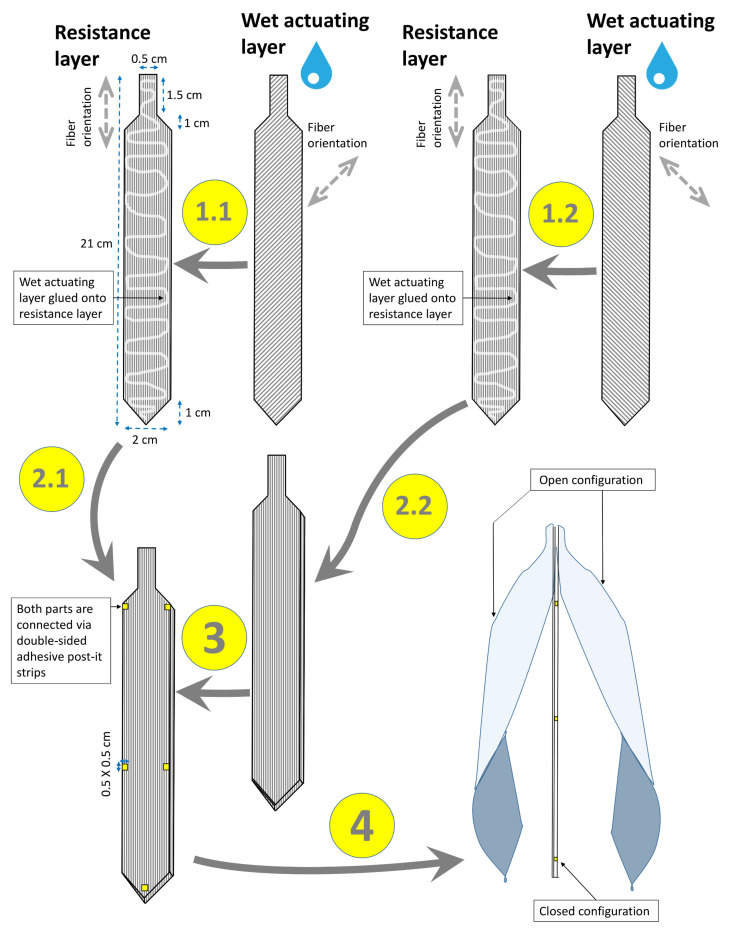
Exemplary construction scheme for the abstraction of the legume (Fabaceae seed pod) and its transfer into a paper-based self-actuated compliant system. The fiber orientations of the dry RLs and wet ALs are indicated (gray dashed arrows). The various construction steps are numbered (1–4). Steps 1.1/1.2 and 2.1/2.2, respectively, can be completed in any order. Details are provided in the main text.

Legume opening and valve twisting can be abstracted and transferred into a paper model via the construction steps depicted in Figure 6. The two valves are constructed as bilayers: the RLs both possess lengthwise fiber orientations, whereas the two wet ALs possess opposite fiber orientations at 45° with respect to their longitudinal axes. Both initially straight valves are then connected at defined areas with double-sided adhesive tape (3M 928 Transferklebeband).

### 2.2. Paper-Polymer Model

For the transfer of the snap-buckling instability of the Venus flytrap (*D. muscipula*), we used the same wet AL paper and glue as indicated in Section 2.1. The polymer sheet was taken by cutting the opaque thicker side of a clip-folder.

#### Snap-Trap of the Carnivorous Venus Flytrap (*D. muscipula*)

**Figure 7:**
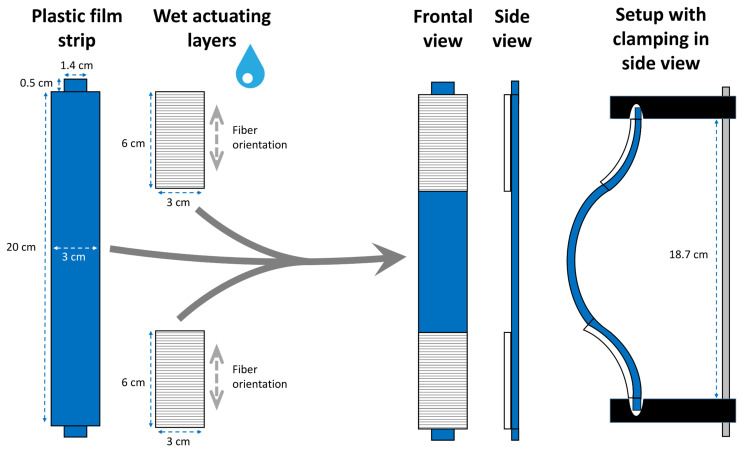
Exemplary construction scheme for the abstraction of snap-through instability as present in the Venus flytrap (*D. muscipula*) and its transfer into a paper-based self-actuated compliant system. The fiber orientations of the wet ALs are indicated (gray dashed arrows). Details are provided in the main text.

The snap-through instability, which speed boosts the trap closure in the Venus flytrap (*D. muscipula*), can be transferred into a paper-polymer model (Figure 7). A strip taken from the polymer sheet acts as the RL and performs the striking curvature inversion, which is initiated by the shrinking processes of two initially wet ALs. The wet ALs are glued on the strip as depicted in Figure 7 so that their fiber orientations are transverse to the longitudinal axis of the strip.

### 2.3. Wood-Polymer Models

A similar desiccation-driven motion actuation involving a coupled bending system has been chosen for the wood-polymer structures that mimic the motion of *A. vesiculosa*. The materials needed for the wood-polymer structure (“Flectofold-like”) are: household string, a clip-folder, and five wooden veneer strips. Six radii of curvature are used as in [43]. The wood-polymer structure is composed of two major substructures: a multilayered backbone composite (wood) and a foldable sheet (polymer). The substructures are manufactured separately and then assembled.

#### Snap-Trap of the Carnivorous Waterwheel Plant (*A. vesiculosa*)

**Figure 8:**
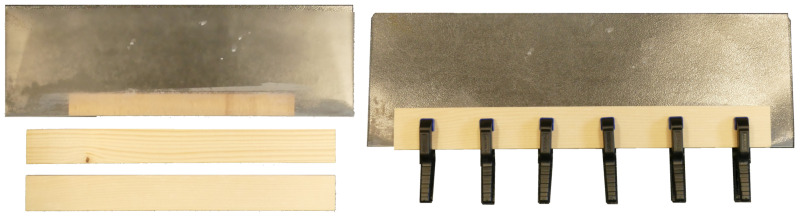
Various steps for clamping the veneer composite by means of boards and clamps in a plastic sleeve.

To produce an actuating multilayered backbone composite, five veneer strips with the dimensions 20 × 190 × 0.5 mm are cut out of a larger sheet of veneer by using a paper cutter. Four veneer strips are cut perpendicular to the grain (actuating zone), and one is cut parallel to the grain (resistance zone). The strips are immersed in water (for approximately 20 s, completely submerged) and are then wiped with a cloth to remove excess water. In the next step, the strips are glued to each other by using water-proof wood glue (Ponal Wasserfest, Henkel, Germany) so as to form a five-layered composite backbone, with the strip cut parallel to the grain being placed on the outside of the composite backbone. The backbone is then clamped between two wooden boards that are slightly larger than the backbone. This ensures that the backbone remains straight during clamping. To prevent the wooden boards from sticking to the wooden strips, a polymer sheet (transparent side of the clip-folder) acts as a “cover” for the boards so that the boards are not in direct contact with the wooden strips (Figure 8). The clamps are then attached to the wooden boards, and the structure is left to set for two hours at room temperature.

**Figure 9:**
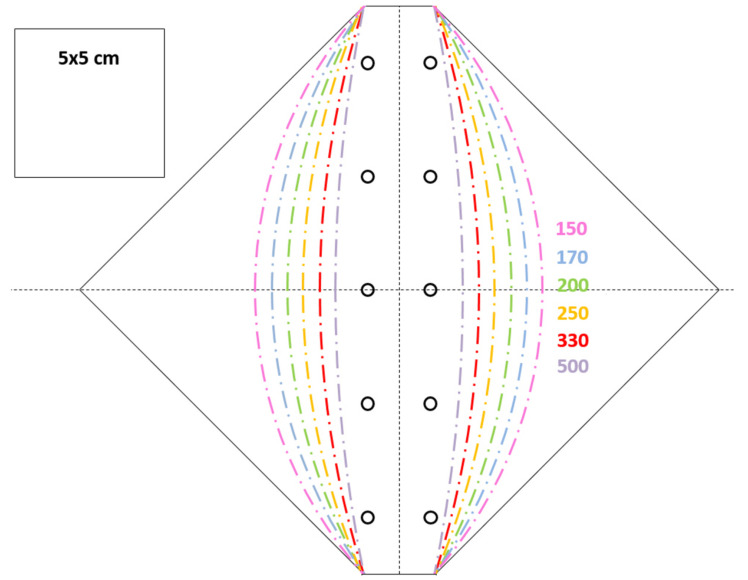
Printable template with six radii of curvature.

The foldable polymer sheet is cut out using a template (Figure 9). This template is printed on paper, cut out, and placed on the rear "hard" side of a clip-folder (Durable Duraclip 30). The rear side is then cut into the same shape as the paper template. One of the six provided radii of curvature (150, 170, 200, 250, 330, 500 mm) on the template can now be chosen. To transfer the curvature from the paper template to the clip-folder sheet, the points on the line of the paper template are pierced with a needle through to the polymer sheet. This creates a hole pattern along the selected curvature lines on the polymer sheet. Furthermore, 10 holes marked on the paper template (to the right and left of the vertical axis) are punched through the polymer sheet by using a 4 mm punching iron. The paper template is then removed, and the clip-folder sheet is folded along the curved lines. The polymer sheet must be folded back and forth from one side to the other about 30 times to achieve certain flexibility along the folding curvature.

**Figure 10:**
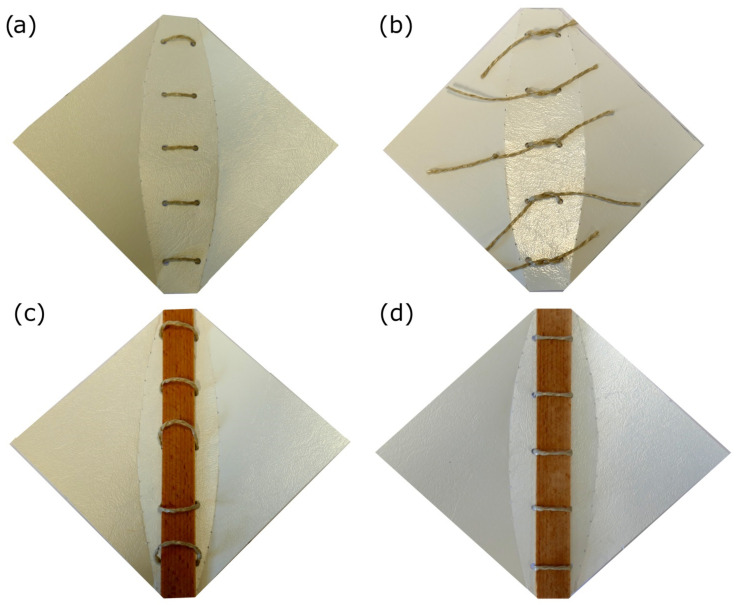
Various steps for connecting the Flectofold-like demonstrator to the actuating multilayer veneer composite. The five pieces of string are threaded through the adjacent large holes in the polymer sheet (**a**), and each is loosely knotted on the back of the demonstrator by an overhand knot (**b**). The wooden veneer is pushed through the string loops (**c**) and firmly attached to the polymer sheet (**d**) by tightening the overhand knots and then multiply knotting the strings with overhand knots on the back of the demonstrator.

Next, the wood-polymer structure is assembled (Figure 10). The clamps are removed from the multilayered veneer backbone, which is then fixed together with the polymer sheet by threading each of five pieces of household string (approximately 20 cm in length) through the pairs of large holes in the clip-folder sheet and knotting each piece of string. The following steps are of importance: (1) the strip cut parallel to the grain is on top, and (2) the triangular side-pieces of the polymer sheet are slightly bent upwards to induce a folding direction.

## 3. Results

In the following, we present examples of solutions for the biomimetic transfer of actuation and/or deformation processes of interest. The geometries and dimensions indicated in the construction “schemes” and the paper and glue types indicated in the Materials and Methods section are not mandatory. Indeed, many models can be geometrically distorted or constructed with other materials (e.g., other paper and glue types) and still show the anticipated movements. However, the presented models all have in common that they function reliably, are easy to assemble, and are small in size. Additionally, the individual materials of the wood-polymer models can be easily separated from each other and disposed of.

### 3.1. Paper-Based Models

#### 3.1.1. Flower of the Bird of Paradise (*S. reginae*)

**Figure 11:**
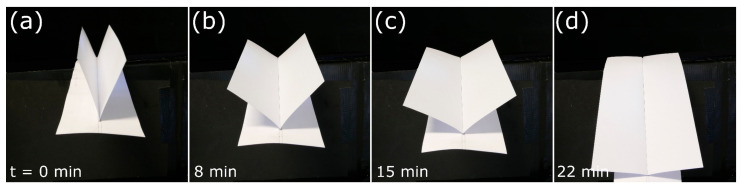
A self-actuated paper-based compliant system inspired by the bird of paradise flower (*S. reginae*). (**a**–**d**) Opening sequence during desiccation. Time is indicated bottom left.

In our tested model (Figure 11, Video S1), the whole drying-induced bending process lasted approximately 22 min. The initially upwards oriented flaps gradually bend laterally downward, as dictated by the increasing curvature of the AR/AL bilayer. Thereby, the whole structure opens out, similar to the natural flower sheath, which opens under the weight force of the pollinating bird.

#### 3.1.2. False Rose of Jericho (*S. lepidophylla*)

**Figure 12:**
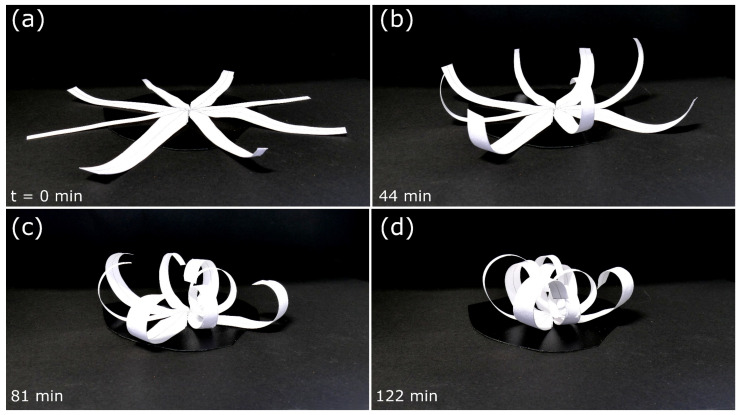
Self-actuated paper-based compliant system inspired by the composite architecture of *S. lepidophylla* performing closure upon drying. (**a**–**d**) Folding sequence during desiccation. Time is indicated bottom left.

We chose two types of “stems” differing in size, with type 1 being smaller than type 2, in our model. Each type was constructed four times. Upon drying, the compliant system showed a striking composite closure motion, with type 1 “stems” curling faster than type 2 (Figure 12, Video S2). The overall motion was completed after 122 min, and a closed state similar to the natural counterpart (cf. Figure 1b) was attained.

#### 3.1.3. Snap-Trap of the Carnivorous Waterwheel Plant (*A. vesiculosa*)

**Figure 13:**
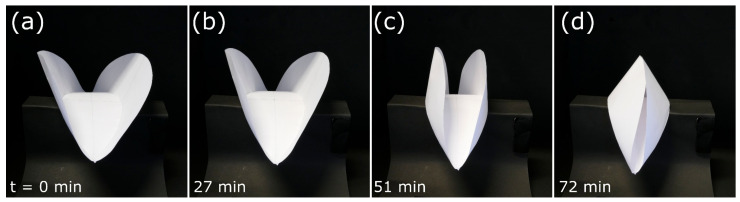
Self-actuated paper-based compliant system inspired by the snap-trap of the waterwheel plant (*A. vesiculosa*) performing closure upon drying. (**a**–**d**) Snapping sequence during desiccation. Times are indicated bottom left.

Full closure of the artificial “trap” in our model occurred within 72 min (Figure 13, Video S3). The lens (rib) progressively bent during the drying process, and the lateral folds were drawn together. In the closed state, the folds (representing the natural trap lobes) pressed against each other.

#### 3.1.4. Blooming of the Lily Flower (*L.* spec.)

**Figure 14:**
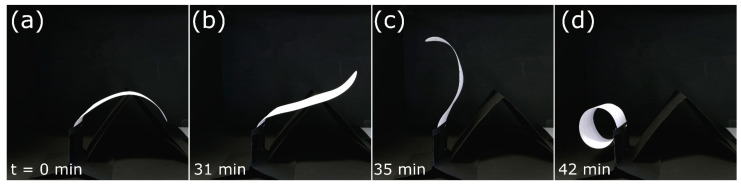
Self-actuated paper-based compliant system inspired by the lily petal (*L.* spec.). (**a**–**d**) Bending sequence during desiccation. Times are indicated bottom left.

The model shows a striking bending deformation during drying, which is completed after 42 min (Figure 14, Video S4) and is highly reminiscent of the blooming motion of the natural petal.

#### 3.1.5. Opening of the Legume

**Figure 15:**
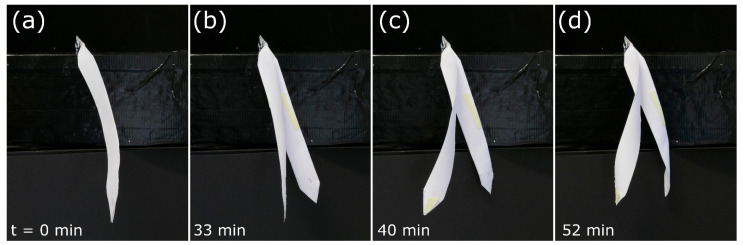
Self-actuated paper-based compliant system inspired by the legume (Fabaceae seed pod) performing rapid opening (at *t* = 33 min) (**a**,**b**) and continuous opposite-handed twisting of the valves during desiccation (**b**–**d**). Times are indicated bottom left.

The drying-induced deformation of the valves in our model induced their rapid splitting (popping) after 33 min (Figure 15, Video S5). In the natural seed pod, this splitting leads to the scattering of the seed. The opposite-handed twisting of the two valves continues until maximum seed pod opening is achieved after 52 min.

### 3.2. Paper-Polymer Model

#### Snap-Trap of the Carnivorous Venus Flytrap (*D. muscipula*)

**Figure 16:**
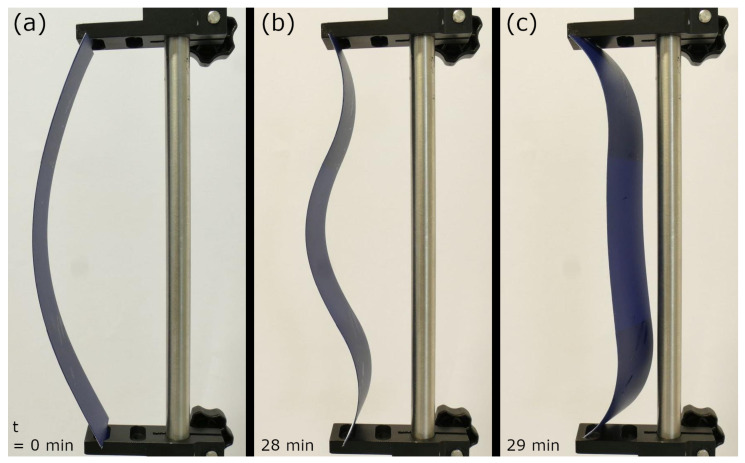
Self-actuated paper-polymer-based compliant system inspired by the Venus flytrap (*D. muscipula*) performing a rapid snap-through transition (at *t* = 29 min). (**a**) Initial state. (**b**) Half-completed movement, shortly before snap-buckling. (**c**) Final state. Times are indicated bottom left.

We used a clamping system to install the initial “outward” curvature of the polymer sheet strip with the two ALs (Figure 16, Video S6) in our model. The drying-induced shrinking of the ALs caused a continuous deformation of the strip until its curvature suddenly flipped “inward” at *t* = 29 min.

### 3.3. Wood-Polymer Models

Snap-Trap of the Carnivorous Waterwheel Plant (*A. vesiculosa*)

**Figure 17:**
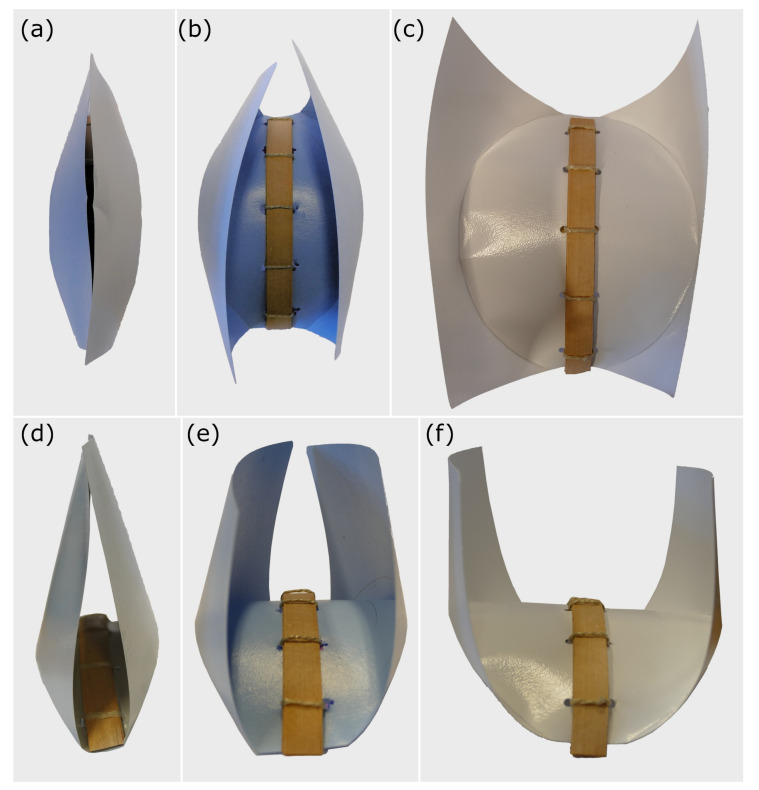
Top and side view of various Flectofold-like structures after the completed movement. The positive correlation between the radius of curvature and the maximal closing angle of the “wings” can be easily discerned. Radius of curvature: (**a**,**d**) 190 mm, (**b**,**e**) 150 mm, and (**c**,**f**) 120 mm.

The bending and folding process of the polymer sheet attributable to the desiccation of the five-layered composite backbone is completed after approximately 24–48 h. Even though the required actuation force cannot be measured, the relationship between the radius of curvature of the fold, the radius of curvature of the wood backbone, and the maximal closing angle during the closing of the Flectofold-like structures can be observed (Figure 17). The closing motion of two structures with a radius of curvature of 200 and 250 mm can be followed in the Supplementary Materials (Video S7) and for two structures with a radius of curvature of 330 and 500 mm (Video S8).

## 4. Discussion

The abstraction of plant movement principles and their transfer into biomimetic 4D printed structures form part of a rapidly progressing research field [10,11,12,13,14,15,16,18,19,20,21,22,23,24,25,26,27,28]. However, 4D printing requires special user skills and expensive hardware hindering its application in research and teaching, which cannot cover such high costs. Our approach offers a low-cost alternate methodology for developing biomimetic self-actuated compliant systems for applications, e.g., in the context of didactic approaches, arts-crafting, and sensors (i.e., humidity). With regard to material costs, each of the paper-based models presented in this paper is in the sub-Euro (€) region and, therefore, affordable for everyone. The models that include polymer foil from clip-holders are only slightly more expensive (in the low €-region). Although the use of paper and wood for handcrafting hygroscopic structures is not new [33], the transfer and successful technical implementation of complex plant deformation principles as presented here have not been previously achieved. We are convinced that many more plant movement and deformation principles can be similarly abstracted and produced. Promising potential concept generators in which the above-described concept can be applied include the hygroscopic seed capsules of the ice plant (*Delosperma nakurense*) [47], the twisting and hook-forming root hairs of English ivy (*Hedera helix*) [48], and the coiling of the awn of the stork’s bill (*Erodium gruinum*) [49].

Most of the hygroscopically actuated models presented in this paper perform desiccation-induced and, thereby, shrinkage-driven movements. Exceptions are the models of the legume (Fabaceae seed pod) and of the snap-trap of *D. muscipula* in which the motion additionally incorporates elastic instabilities. Thus, complex combinations of various movement actuation principles can indeed be achieved with simple manual construction methods.

With regard to the lily petal structure (Figure 5 and Figure 14, Video S4), we must state that, in the natural flower, expansion (growth) of the outer petal zone (edge) takes place, which is accompanied by petal wrinkling. However, despite numerous attempts, we have not been able to create a similar marginal zone on the artificial “petal”. Therefore, the marginal zone in our model, which is constructed as a functional bilayer, represents an additional abstraction step leading to the observed motion.

Whereas several concept generators used in this study show repeatable motions (*S. reginae* flower, *S. lepidophylla* stems, snap-traps of *A. vesiculosa* and *D. muscipula*), some other processes (slow *Lilium* flower opening, rapid Fabaceae seed pod opening) are not repeatable, and the respective structures, thereby, constitute “single-use” devices (in the context of the performed motions). Due to structural disintegration during selective rewetting of the actuating layers, the presented paper-based models also function only once, whereas the paper-polymer model (at least to some extent) and, specifically, the wood-polymer models can be actuated numerous times. The Flectofold-like structures (Figure 17) can be examined during the process of reopening during drying. Therefore, such more expensive and elaborate models have a reversibility feature as an extra.

Our models were not developed to act as “product demonstrators” or similar milestones in research and development approaches. This can only be achieved by precise additive manufacturing and other reliable methods. Instead, our models should be regarded as (1) easy-to-achieve functional demonstrators for the proof-of-concept of certain functionalities (e.g., the interplay of structure and motion as revealed here), and (2) as a means for teaching the “manual comprehension” of plant structure, mechanics, and movement. In particular, the low constructional costs and the easy and time-saving manufacturing procedures allow the production of numerous physical models and model generations. In the context of teaching, students are able to develop technical counterparts of motile plant structures and to demonstrate physically correct (or abstracted) movement actuation and/or deformation processes by such modeling. The involved processes of abstracting biological working principles and their transfer into technical materials require a wealth of ideas and spatial imagination on the part of the students who can either follow a set of instructions (such as the construction schemes presented here) or their own inspiration. This has an enormously motivating effect on learning and creates a deeper understanding of fundamental mechanical principles both in a plant-inspired technical application (biomimetics) and in the biological model (reverse biomimetics).

## Data Availability

All relevant data are included within the paper and its Supplementary Materials files.

## Supplementary Materials

The following are available online at https://www.mdpi.com/article/10.3390/biomimetics6030042/s1, Video S1: A self-actuated paper-based compliant system inspired by the bird of paradise flower (*S. reginae*), playback rate: 10 fps (100× time lapse), Video S2: A self-actuated paper-based compliant system inspired by the composite architecture of *S. lepidophylla*, playback rate: 10 fps (100× time lapse), Video S3: A self-actuated paper-based compliant system inspired by the snap-trap of the waterwheel plant (*A. vesiculosa*), playback rate: 10 fps (100× time lapse), Video S4: A self-actuated paper-based compliant system inspired by the lily petal (*L.* spec.), playback rate: 10 fps (100× time lapse), Video S5: A self-actuated paper-based compliant system inspired by the legume fruit (Fabaceae seed pod), playback rate: 10 fps (100× time lapse), Video S6: A self-actuated paper-polymer-based compliant system inspired by the Venus flytrap (*D. muscipula*), playback rate: 10 fps (100× time lapse), Video S7: Closing motion of a wood-polymer-based Flectofold structure with 200 und 250 mm radius of curvature, playback rate: 10 fps (2400× time lapse), Video S8: Closing motion of a wood-polymer-based Flectofold structure with 330 und 500 mm radius of curvature, playback rate: 10 fps (2400× time lapse). Videos with higher resolution can be obtained from the authors upon request.
